# The Effects of Bone‐Remodeling Therapy on Survival, Pain, and Skeletal Related Events in the Setting of Renal Cell Carcinoma With Bone Metastases: A Multicenter Investigation From a Large Global Health Research Network (TriNetX)

**DOI:** 10.1002/cam4.71133

**Published:** 2026-01-14

**Authors:** Brian H. Im, Kevin K. Zarrabi, Aaron R. Hochberg, Mihir S. Shah, James R. Mark, Joseph K. Izes, Patrick T. Gomella, Costas D. Lallas, Leonard G. Gomella, Adam R. Metwalli

**Affiliations:** ^1^ Department of Urology, Sidney Kimmel Medical College Thomas Jefferson University Philadelphia Pennsylvania USA; ^2^ Department of Medical Oncology, Sidney Kimmel Medical College Thomas Jefferson University Philadelphia Pennsylvania USA

**Keywords:** bone metastases, BPs, chronic pain, overall survival, RANK‐L inhibitors, renal cell carcinoma

## Abstract

**Introduction:**

Renal cell carcinoma (RCC) is the most common renal malignancy—bone metastases (BM) are indicative of aggressive disease with a poor prognosis. We aim to evaluate the overall mortality of patients with RCC with and without BM, and to elucidate the effects of bone‐remodeling therapy on mortality, incidence of skeletal‐related events, and opioid usage patterns in patients with BM.

**Methods:**

A retrospective cohort study was conducted using TriNetX, a large, collaborative network sourced from electronic medical records of over 110 million patients from over 100 healthcare organizations. All adult patients with RCC and RCC with BM were queried.

**Results:**

There were 139,859 patients diagnosed with RCC, of which 9021 had RCC with bone metastases (BM). Among these, 999 patients received only bisphosphonates (BPs), 973 received only RANK ligand inhibitors (RANKLi), and 119 patients initially received BPs before switching to RANKLi.

Presence of BM results in a ~2.5‐fold increase in 5‐year mortality rate (*p* < 0.0001) and a statistically significant increase in all SREs compared to those without BM. Patients receiving bisphosphonates had significantly higher rates of opioid use (*p* < 0.001) and comparable rates of chronic pain diagnoses to the overall BM group (*p* = 0.9217). In contrast, patients receiving RANKLi had a statistically significant reduction in opioid use (*p* < 0.001) and hypocalcemia (*p* < 0.001) compared to those on BPs, and an improved 5‐year survival rate (*p* < 0.0001) and median survival (*p* < 0.0001) relative to the overall BM cohort.

**Conclusion:**

Patients receiving RANKLi have significantly improved survival, reduced opioid use and lower rates of SREs. Patients with RCC and BMs experienced significantly worse outcomes compared to those without BM. Surprisingly, among RCC patients with BMs, those treated with BPs only experienced even poorer outcomes.

## Graphical abstract

In this retrospective population study containing 139,859 patients, subjects receiving RANK‐ligand inhibitors had improved survival and decreased rates of skeletal‐related events relative to the overall cohort of patients with metastatic bone lesions. Bone‐modifying agents may confer improved survival rates and decreased rates of skeletal‐related events.

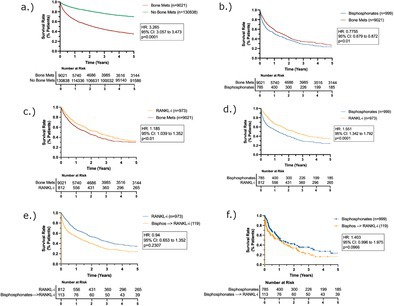

## 1 Introduction

Renal cell carcinoma (RCC) is the most common renal malignancy, accounting for over 90% of cancers originating in the kidney. RCC is more common in men than women, most often diagnosed in adults 60–70 years of age [1]. Bone metastases (BM) are the second most common site of metastasis for RCC after the lungs [2]. The pathogenesis of BM begins with metastasis‐induced osteoclast hyperactivity, leading to lytic bone lesions and the secretion of several bone‐derived growth factors and cytokines, including transforming growth factor‐beta (TGF‐B) and fibroblast growth factor (FGF), which perpetuate metastatic growth and additional bone lesions [3]. These metastatic bone lesions interact with the Receptor Activator of Nuclear factor Kappa‐Β Ligand **(**RANKL) system, resulting in significant aberrations in calcium homeostasis and bone integrity [4, 5]. Presence of BM is indicative of aggressive disease and poor prognosis, largely in part due to skeletal‐related events (SRE), including bone pain, pathologic fractures, compressive neuropathies, hypercalcemia, and hypocalcemia [6, 7]. BM‐induced SREs are associated with significant pain, measurable quality‐of‐life deficits, and increased mortality [8, 9].

To date, there are no large‐scale studies evaluating the effects of bone‐remodeling therapy (bisphosphonates and/or RANKL inhibitors) in the setting of metastatic RCC with BMs. We aim to assess the morbidity and mortality in patients with RCC with and without BM. Additionally, we aim to explore the impact of bone‐remodeling therapy on SREs, chronic pain diagnosis and opioid use patterns, and overall survival rates in patients with BM. We hypothesize that overall mortality, chronic pain diagnoses, and opioid usage rates are higher in those with BM compared to those without BM. Additionally, we hypothesize that bone‐remodeling therapy confers a reduction in SREs and opioid usage, and an overall improved mortality rate.

## 2 Methods

### 2.1 Data Source

We utilized the TriNetX database, a large, collaborative network containing data from electronic medical records (EMR) from over 110 million patients sourced from over 100 healthcare organizations (HCO), to generate our patient cohorts. TriNetX contains patient data from up to 20 years prior to the date of generation of the query (2005–2025). Once data sourced from the HCOs is imported into TriNetX, an extensive quality assessment is conducted, ensuring a certain standard of quality is met with the dataset. This study is exempt from ethical approval and is de‐identified to standards defined in *Section §164.514(a)* of the HIPAA Privacy Rule. This process was attested to by a qualified expert, defined in *Section §164.514(b)* (1) of the HIPAA Privacy Rule.

### 2.2 Study Population

**FIGURE 1:**
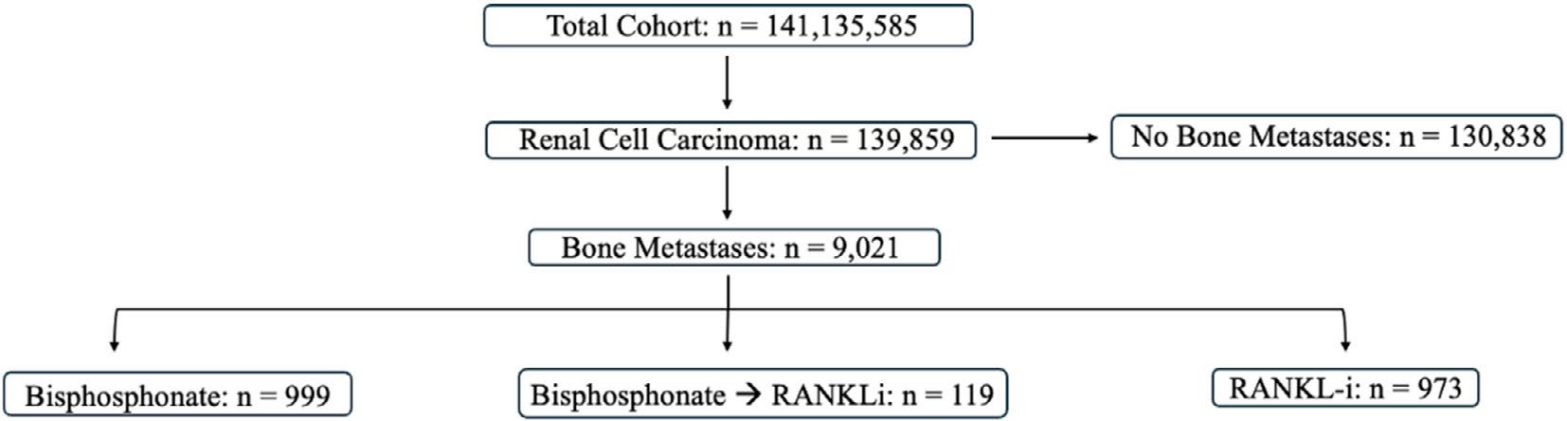
Consort diagram for patient selection.

The selection methods of the study population are detailed in Figure 1. We initially queried for all adult patients with a diagnosis of renal cell carcinoma, using the following ICD‐10 codes: C64.1 (malignant neoplasm of right kidney, excluding renal pelvis) and C64.2 (malignant neoplasm of left kidney, excluding renal pelvis). Then, we queried all patients with RCC with a subsequent diagnosis of BM using the ICD‐10 code C79.51 (secondary malignant neoplasm of bone). From there, patients who were prescribed the following bone‐remodeling agents following a diagnosis of either RCC or RCC complicated by BM were queried: bisphosphonates (BPs) (zoledronate, ibandronate, risedronate, alendronate, etidronate, pamidronate) and RANKL inhibitors (RANKLi) (denosumab). Additionally, patients with BM who were initially prescribed BPs then subsequently prescribed RANKLi were queried (BP➔RANKLi). Patients receiving the following opioids were identified: hydrocodone, hydromorphone, morphine, codeine, tramadol, oxycodone, and naloxone (which acts as a proxy for opioid prescription).

### 2.3 Outcomes and Statistical Analysis

Our primary outcome was patient mortality. Our secondary outcomes included rates of chronic pain diagnoses, opioid usage patterns, rates of vertebroplasty and other orthopedic interventions (Repair, Revision, and/or Reconstruction Procedures on the Pelvis and Hip Joint [MLS:CPT:1004897] or Fracture and/or Dislocation Procedures on the Pelvis and Hip Joint [UMLS:CPT:1004933]), and rates of palliative radiation treatment. The index event was a diagnosis of RCC with or without BM, as well as the prescription of bone‐remodeling therapy.

Our primary comparison was patients with BM to patients taking either BPs or RANKLi. Secondary comparisons included patients with BM to those without BM, those with BM taking BPs or RANKLi‐only to those with BM not taking either, those taking BP‐only to those taking RANKLi‐only, and those taking BPs who were subsequently prescribed RANKLi to those prescribed either BPs or RANKLi only. These were selected to assess the mortality rates of RCC complicated by BM against that of patients without BM, as well as to compare the therapeutic efficacy of BPs to that of RANKLi.

We conducted propensity‐score matching (PSM) to limit confounding variables in our comparisons. TriNetX has integrated PSM directly into the user interface, allowing users to generate ICD‐10/CPT‐based queries for use as parameters of interest. We matched all cohorts for the following parameters: age, gender, BMI, CKD‐stage/ESRD, nephrectomy status (radical, partial), histology, staging, external beam radiation therapy (EBRT), chemotherapy regimen (axitinib, pembrolizumab, cabozantinib, nivolumab, lenvatinib, ipilimumab, everolimus, sunitinib, sorafenib, belzutifan, tivozanib), and osteoporosis status.

We conducted Cox proportional hazards model analysis to further parse the effect of various covariates on both 5‐year mortality and opioid usage patterns in each of our cohorts. The following covariates were included: male gender, age, CKD status (N18: Chronic kidney disease (CKD)), cancer staging, nephrectomy status (Z90.5: acquired absence of kidney), EBRT, liver mets (C78.7: Secondary malignant neoplasm of liver and intrahepatic bile duct), lung mets (C78.0: Secondary malignant neoplasm of lung), brain mets (C79.31: Secondary malignant neoplasm of brain), and positive lymph nodes (C77: Secondary and unspecified malignant neoplasm of lymph nodes).

All statistical analysis was conducted internally using the TriNetX database's built‐in analysis functions. For our primary outcomes, we generated Kaplan–Meier survival curves, reporting median survival, log‐rank test results, and odds ratios (OR) and hazard ratios (HR) with 95% confidence intervals (CI). For our secondary outcomes, we reported odds ratios with 95% CI. Cox proportional hazard models reported as hazard ratios (HR) with 95% CI. For all statistical studies, *p* < 0.05 was considered statistically significant.

## 3 Results

### 3.1 Patient Demographics and Mortality

**FIGURE 2:**
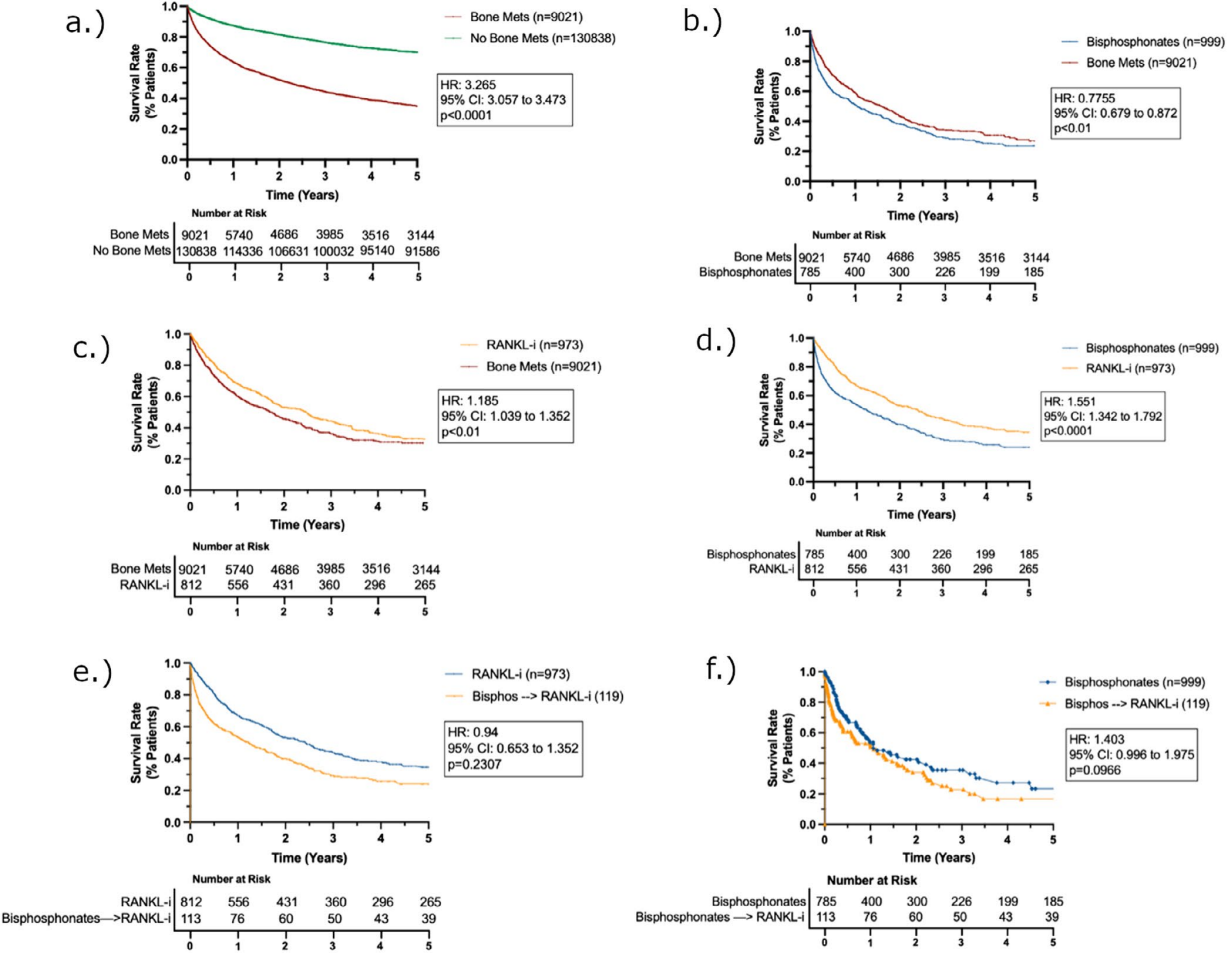
Overall survival (OS); Kaplan–Meier estimates for OS in (a) patients with and without BMs; (b) patients with BMs treated with or without BPs; (c) patients with BMs treated with or without RANKLi; (d) patients with BMs treated with either BPs or RANKLi; (e) patients with BMs treated with RANKLi or BP ➔ RANKLi; (f) patients with BMs treated with BP or BP ➔ RANKLi. KM analyses performed with propensity score weighting.

There were 139,859 patients with a diagnosis of RCC. There were 9021 patients with RCC + BM. 999 patients received only BPs. 973 patients received only RANKLi. 119 patients initially received BPs; then subsequently received RANKLi. (Table 1). 5‐year survival and Kaplan–Meier survival analyses for all cohorts are shown in Figure 2a–f and Table 2.

**TABLE 1:** Baseline patient demographics.

	RCC with BM, *N* (%)	RCC with no BM, *N* (%)	*p*	RCC with BM + BPs, *N* (%)	*p*	RCC with BM + RANKLi, *N* (%)	*p*	RCC with BM + BPs ➔ RANKLi, *N* (%)	*p*
Number of Patients	9021	130,838	< 0.0001	999	—	973	—	119	—
Mean Age at Diagnosis	65.9	62.9	< 0.0001	64.3	< 0.0001	66	0.9901	67.7	0.0046
Sex: Male	5218 (66.1%)	67,058 (59.8%)	< 0.0001	508 (64.7%)	0.3179	490 (64.6%)	0.2277	75 (66.4%)	0.8702
Female	2274 (28.8%)	40,830 (36.4%)	< 0.0001	236 (30%)	0.3776	223 (29.4%)	0.5245	33 (29.2%)	0.8635
Unknown	407 (5.2%)	4350 (3.9%)	< 0.0001	41 (5.2%)	0.7337	46 (6.1%)	0.1848	10 (8.8%)	0.4828
Nephrectomy status: partial	219 (3%)	2927 (3%)	< 0.0001	32 (4%)	0.01	22 (3%)	0.01	6 (5.3%)	0.9435
Nephrectomy status: radical	1277 (14%)	4077 (3%)	< 0.0001	116 (15%)	0.9340	118 (16%)	0.05	18 (15.9%)	0.5312

Abbreviations: BM, metastatic bone lesions; BP, bisphosphonates; RANKLi, RANK ligand inhibitors; RCC, renal cell carcinoma.

**TABLE 2:** 5‐year survival data for all cohorts.

	5‐year survival (%)	Median survival (Months)	Hazard ratio (HR) (95% CI)	*p*
A. Survival compared to all RCC with BM				
RCC with BM	31.50%	28.1	—	—
RCC with no BM	83.30%	220.0	3.108 (2.924, 3.303)	< 0.0001
RCC with BM + BPs	24.80%	16.0	1.224 (1.072, 1.397)	< 0.01
RCC with RANKLi	34.00%	29.6	0.796 (0.693, 0.915)	< 0.01
B. Survival Compared to RCC with BP				
RCC with BP	31.50%	16.0	—	—
RCC with RANKLi	83.30%	220.0	0.628 (0.535, 0.738)	< 0.0001
RCC with BP – > RANKLi	26.90%	20.4	0.804 (0.562, 1.15)	0.1318
C. Survival Compared to RCC with RANKLi				
RCC with RANKLi	83.30%	220.0	—	—
RCC with BP – > RANKLi	26.90%	20.4	1.338 (0.948, 1.891)	0.0966

Abbreviations: BM, metastatic bone lesions; BP, bisphosphonates; RANKLi, RANK ligand inhibitors; RCC, renal cell carcinoma.

Presence of BM results in a ~2.5‐fold increase in 5‐year mortality rate (83.3% vs. 31.5%, HR: 3.108, 95% CI: 2.924 to 3.303, log‐rank *p* < 0.0001) compared to those without BM. Additionally, those with BM were over 4‐times more likely to have received a radical nephrectomy compared to those without BM (14% vs. 3%, *p* < 0.0001), and over 3‐times as likely to have received a partial nephrectomy (1.7% vs. 0.5%, *p* < 0.0001). However, the cohort of patients receiving BPs had a statistically significantly lower 5‐year survival (24.8% vs. 31.5%, HR 1.22, 95% CI: 1.07 to 1.39, log‐rank *p* < 0.001) and median survival (16.0 months vs. 28.1 months, *p* < 0.001) compared to our overall BM cohort.

The cohort receiving RANKLi‐only, on the other hand, had a significant increase in both 5‐year survival (34.0% vs. 24.8%, HR: 0.628, 95% CI: 0.535 to 0.738, *p* < 0.0001) and median survival (29.6 months vs. 16.0 months, *p* < 0.0001) relative to the BP cohort, as well as an improved 5‐year mortality rate relative to the overall BM group (34.0% vs. 31.5%, HR: 0.796, 95% CI: 0.693 to 0.915, log‐rank *p* < 0.01).

Notably, patients in the BP → RANKLi cohort exhibited slightly lower 5‐year survival rates (26.9% vs. 34.0%, *p* = 0.1318) and median survival times (20.4 months vs. 29.6 months) compared to those who received RANKLi‐only. In contrast, when compared to the cohort receiving BP‐only, the BP ➔ RANKLi group showed a trend toward higher 5‐year survival rates (26.9% vs. 24.8%, *p* = 0.0966); though this difference also did not reach statistical significance.

Hazard ratios for all Cox proportional hazard model covariates affecting 5‐year mortality, 95% CI, and *p*‐values are shown in Table 3. Cox proportional models comparing the BM cohort to the no BM cohort showed that CKD status, cancer clinical stages 3 and 4, EBRT, and presence of metastatic lesions significantly contributed to lower mortality, while nephrectomy status was protective. When comparing the BM cohort to the BP‐only cohort, the presence of additional metastatic lesions largely contributed to the greater mortality in the BP cohort; nephrectomy status continued to be protective. Notably, when comparing the RANKLi‐only cohort to the BP➔RANKLi cohort, CKD status and the presence of liver mets significantly diminished 5‐year survival.

**TABLE 3:** Cox proportional hazards of mortality associated with bone‐remodeling therapy.

	RCC + BM vs. No BM		RCC + BM vs. BP		RCC + BM vs. RANKLi		RCC + BM vs. RANKLi		RCC + RANKLi vs. BP➔RANKLi		RCC + BP vs. BP➔RANKLi	
	HR (95% CI)	*p*	HR (95% CI)	*p*	HR (95% CI)	*p*	HR (95% CI)	*p*	HR (95% CI)	*p*	HR (95% CI)	*p*
Male Gender	1.104 (0.852, 0.963)	< 0.0001	0.906 (0.852, 0.963)	0.0016	0.907 (0.853, 0.965)	0.002	—	—	0.991 (0.831, 1.182)	0.9189	—	—
Age	1.039 (1.005, 1.01)	< 0.0001	1.008 (1.005, 1.01)	< 0.0001	1.008 (1.005, 1.01)	< 0.0001	—	—	0.997 (0.989, 1.005)	0.433	—	—
CKD	1.518 (1.146, 1.309)	< 0.0001	1.225 (1.146, 1.309)	< 0.0001	1.222 (1.143, 1.306)	< 0.0001	—	—	1.224 (1.021, 1.466)	0.0285	—	—
Tumor Staging: S1	0.854 (0.826, 1.15)	< 0.0001	0.975 (0.826, 1.15)	0.7625	0.885 (0.748, 1.048)	0.1571	—	—	0.609 (0.356, 1.039)	0.0688	—	—
Tumor Staging: S2	1.055 (0.84, 1.266)	0.3611	1.031 (0.84, 1.266)	0.7711	1.028 (0.841, 1.256)	0.7909	—	—	1.388 (0.897, 2.148)	0.1406	—	—
Tumor Staging: S3	1.324 (0.915, 1.249)	< 0.0001	1.069 (0.915, 1.249)	0.3987	1.063 (0.913, 1.238)	0.432	—	—	1.07 (0.734, 1.561)	0.7243	—	—
Tumor Staging: S4	2.292 (1.014, 1.264)	< 0.0001	1.132 (1.014, 1.264)	0.0271	1.2 (1.077, 1.338)	0.001	—	—	0.997 (0.763, 1.304)	0.9853	—	—
Nephrectomy	0.71 (0.673, 0.77)	0.0038	0.72 (0.673, 0.77)	< 0.0001	0.725 (0.678, 0.776)	< 0.0001	—	—	0.773 (0.645, 0.927)	0.0054	—	—
EBRT	2.499 (0.881, 5.109)	< 0.0001	2.121 (0.881, 5.109)	0.0935	1.714 (0.642, 4.577)	0.2824	—	—	—	—	—	—
Liver Mets	1.629 (1.255, 1.502)	< 0.0001	1.373 (1.255, 1.502)	< 0.0001	1.419 (1.296, 1.555)	< 0.0001	—	—	1.714 (1.397, 2.104)	< 0.0001	—	—
Lung Mets	1.34 (1.086, 1.249)	< 0.0001	1.165 (1.086, 1.249)	< 0.0001	1.16 (1.081, 1.244)	< 0.0001	—	—	1.157 (0.97, 1.381)	0.1048	—	—
Brain Mets	1.387 (1.134, 1.38)	< 0.0001	1.251 (1.134, 1.38)	< 0.0001	1.22 (1.103, 1.349)	< 0.0001	—	—	1.268 (0.997, 1.611)	0.0526	—	—
Lymph Nodes	1.539 (1.31, 1.514)	< 0.0001	1.408 (1.31, 1.514)	< 0.0001	1.359 (1.262, 1.463)	< 0.0001	—	—	1.223 (1.015, 1.474)	0.0341	—	—

### 3.2 Skeletal Related Events and Drug‐Related Adverse Effects

Incidence rates for SREs and hypercalcemia/hypocalcemia, odds ratios (OR), 95% CI, and *p*‐values are shown in Tables 4, 5, 6, 7, 8, 9 and Tables 10, 11, 12, 13, 14, 15. The RCC + BM patient cohort had a statistically significant increase in the incidence of all SREs compared to those without BM (Tables 4, 5, 6, 7, 8, 9) in addition to higher rates of hypercalcemia (14.2% vs. 5.4%, OR: 2.917, 95% CI: 2.595 to 3.279, *p* < 0.0001) and hypocalcemia (7.4% vs. 3.2%, OR: 2.458, 95% CI: 2.11 to 2.863, *p* < 0.0001). Additionally, relative to those receiving BP‐only, patients in the RANKLi cohort had significantly lower rates of hypercalcemia (40.4% vs. 13.8%, OR: 4.253, 95% CI: 3.193 to 5.663, *p* < 0.0001), while the BP➔ RANKLi cohort had non‐significantly lower rates of hypercalcemia (42.1% vs. 34.6%, OR: 1.373, 95% CI: 0.79 to 2.388, *p* = 0.2606).

**TABLE 4:** Comparison of skeletal‐related event (SRE) rates associated with bone‐remodeling therapy between BM cohort vs. No BM cohort.

Complications of bone‐remodeling therapy				
	RCC + BM	RCC + No BM	OR (95% CI)	*p*
Chronic pain	50.1%	27.2%	2.686 (2.512, 2.871)	< 0.0001
Opioid use	80.8%	71.8%	1.65 (1.531, 1.778)	< 0.0001
Vertebroplasty	6.6%	0.6%	12.7 (9.289, 17.364)	< 0.0001
Orthopedic intervention	3.2%	0.9%	3.664 (2.801, 4.794)	< 0.0001
Bone‐directed radiation treatment	31.4%	8.0%	5.221 (4.75, 5.739)	< 0.0001

Abbreviations: BM, metastatic bone lesions; RCC, renal cell carcinoma.

**TABLE 5:** Comparison of skeletal‐related event (SRE) rates associated with bone‐remodeling therapy between BM cohort vs. Bisphosphonate cohort.

Complications of bone‐remodeling therapy				
	BP‐only	RCC + BM	OR (95% CI)	*p*
Chronic pain	54.2%	54.5%	1.0 (0.817, 1.201)	0.9217
Opioid use	92.2%	84.7%	2.145 (1.564, 2.94)	< 0.001
Vertebroplasty	4.8%	5.4%	0.883 (0.57, 1.368)	0.5777
Orthopedic intervention	2.2%	3.4%	0.635 (0.348, 1.157)	0.635
Bone‐directed radiation treatment	33.4%	36.3%	0.881 (0.72, 1.078)	0.2178

Abbreviations: BM, metastatic bone lesions; BP, bisphosphonates; RCC, renal cell carcinoma.

**TABLE 6:** Comparison of skeletal‐related event (SRE) rates associated with bone‐remodeling therapy between BM cohort vs. RANKLi cohort.

Complications of bone‐remodeling therapy				
	RANKLi‐only	RCC + BM	OR (95% CI)	*p*
Chronic pain	57.9%	54.1%	1.168 (0.96, 1.422)	0.1206
Opioid use	84.8%	85.2%	0.971 (0.739, 1.276)	0.8346
Vertebroplasty	6.2%	6.7%	0.921 (0.619, 1.371)	0.6851
Orthopedic intervention	2.5%	3.3%	0.734 (0.408, 1.32)	0.3001
Bone‐directed radiation treatment	40.3%	39.6%	1.031 (0.845, 1.258)	0.7607

Abbreviations: BM, metastatic bone lesions; RANKLi, RANK ligand inhibitors; RCC, renal cell carcinoma.

**TABLE 7:** Comparison of skeletal‐related event (SRE) rates associated with bone‐remodeling therapy between bisphosphonate cohort vs. RANKLi cohort.

Complications of bone‐remodeling therapy				
	RANKLi‐only	BP‐only	OR (95% CI)	*p*
Chronic pain	50.8%	55.9%	0.813 (0.651, 1.016)	0.069
Opioid use	84.8%	90.8%	0.567 (0.396, 0.812)	< 0.001
Vertebroplasty	6.6%	5.0%	1.369 (0.835, 2.246)	0.2115
Orthopedic intervention	2.6%	2.6%	1.0 (0.484, 2.065)	1.000
Bone‐directed radiation treatment	40.6%	33.2%	1.371 (1.081, 1.739)	< 0.01

Abbreviations: BP, bisphosphonates; RANKLi, RANK ligand inhibitors.

**TABLE 8:** Comparison of skeletal‐related event (SRE) rates associated with bone‐remodeling therapy between RANKLi cohort vs. Bisphosphonate ➔ RANKLi cohort.

Complications of bone‐remodeling therapy				
	RANKLi‐only	BP ➔ RANKLi	OR (95% CI)	*p*
Chronic pain	66.7%	70.4%	0.842 (0.474, 1.497)	0.5579
Opioid use	87.9%	81.4%	1.661 (0.78, 3.357)	0.1855
Vertebroplasty	N/A			
Orthopedic intervention	N/A			
Bone‐directed radiation treatment	38.9%	37.0%	1.082 (0.624, 1.875)	0.7792

Abbreviations: BP, bisphosphonates; RANKLi, RANK ligand inhibitors.

**TABLE 9:** Comparison of skeletal‐related event (SRE) rates associated with bone‐remodeling therapy between bisphosphonate cohort vs. Bisphosphonate‐RANKLi cohort.

Complications of bone‐remodeling therapy				
	BP‐only	BP ➔ RANKLi	OR (95% CI)	*p*
Chronic pain	51.4%	68.2%	0.493 (0.282, 0.859)	0.5579
Opioid use	91.6%	81.3%	2.503 (1.083, 5.787)	< 0.05
Vertebroplasty	N/A			
Orthopedic intervention	N/A			
Bone‐directed radiation treatment	31.8%	38.3%	0.75 (0.427, 1.317)	0.3159

Abbreviations: BP, bisphosphonates; RANKLi, RANK ligand inhibitors.

**TABLE 10:** Comparison of adverse outcomes associated with bone‐remodeling therapy between BM cohort vs. no BM cohort.

Complications of bone‐remodeling therapy				
	RCC + BM	RCC + No BM	OR (95% CI)	*p*
Osteonecrosis of Jaw	0.4%	0.1%	2.706 (1.309, 5.595)	< 0.001
Anaphylaxis	N/A			
Hypercalcemia	14.2%	5.4%	2.917 (2.595, 3.279)	< 0.0001
Hypocalcemia	7.4%	3.2%	2.458 (2.11, 2.863)	< 0.0001

Abbreviations: BM, metastatic bone lesions; BP, bisphosphonates; RANKLi, RANK ligand inhibitors; RCC, renal cell carcinoma.

**TABLE 11:** Comparison of adverse outcomes associated with bone‐remodeling therapy between BM cohort vs. BP cohort.

Complications of bone‐remodeling therapy				
	BP‐only	RCC + No BM	OR (95% CI)	*p*
Osteonecrosis of Jaw	N/A			
Anaphylaxis	N/A			
Hypercalcemia	41.1%	22.6%	2.383 (1.926, 2.948)	< 0.0001
Hypocalcemia	9.6%	7.9%	1.235 (0.878, 1.736)	0.2252

Abbreviations: BM, metastatic bone lesions; BP, bisphosphonates; RCC, renal cell carcinoma.

**TABLE 12:** Comparison of adverse outcomes associated with bone‐remodeling therapy between BM cohort vs. RANKLi cohort.

Complications of bone‐remodeling therapy				
	RANKLi‐only	RCC + BM	OR (95% CI)	*p*
Osteonecrosis of Jaw	N/A			
Anaphylaxis	N/A			
Hypercalcemia	13.7%	15.7%	0.862 (0.654, 1.136)	*p* < 0.0001
Hypocalcemia	17.1%	11.2%	1.634 (1.235, 2.186)	*p* < 0.001

Abbreviations: BM, metastatic bone lesions; RANKLi, RANK ligand inhibitors; RCC, renal cell carcinoma.

**TABLE 13:** Comparison of adverse outcomes associated with bone‐remodeling therapy between bisphosphonate cohort vs. RANKLi cohort.

Complications of bone‐remodeling therapy				
	BP‐only	RANKLi‐only	OR (95% CI)	*p*
Osteonecrosis of Jaw	N/A			
Anaphylaxis	N/A			
Hypercalcemia	40.4%	13.8%	4.253 (3.193, 5.663)	*p* < 0.0001
Hypocalcemia	10.2%	16.8%	0.561 (0.398, 0.791)	*p* < 0.001

Abbreviations: BP, bisphosphonates; RANKLi, RANK ligand inhibitors.

**TABLE 14:** Comparison of adverse outcomes associated with bone‐remodeling therapy between RANKLi cohort vs. Bisphosphonate ➔ RANKLi cohort.

Complications of bone‐remodeling therapy				
	RANKLi‐only	BP➔RANKLi	OR (95% CI)	*p*
Osteonecrosis of Jaw	N/A			
Anaphylaxis	N/A			
Hypercalcemia	14.8%	37.0%	0.296 (0.153, 0.572)	*p* < 0.0001
Hypocalcemia	15.7%	13.9%	1.158 (1.298, 2.611)	0.7017

Abbreviations: BP, bisphosphonates; RANKLi, RANK ligand inhibitors.

**TABLE 15:** Comparison of adverse outcomes associated with bone‐remodeling therapy between bisphosphonate cohort vs. bisphosphonate ➔ RANKLi cohort.

Complications of bone‐remodeling therapy				
	BP‐only	BP➔RANKLi	OR (95% CI)	*p*
Osteonecrosis of Jaw	N/A			
Anaphylaxis	N/A			
Hypercalcemia	42.1%	34.6%	1.373 (0.79, 2.388)	0.2606
Hypocalcemia	11.2%	14.0%	0.775 (0.344, 1.744)	0.5368

Abbreviations: BP, bisphosphonates; RANKLi, RANK ligand inhibitors.

However, the RANKLi patient subset had a significantly higher rate of hypocalcemia relative to the overall BM cohort (17.1% vs. 11.2%, OR: 1.63, 95% CI: 1.23 to 2.18, *p* < 0.001). Similarly, the BP only cohort (10.2% vs. 16.8%, OR: 0.561, 95% CI: 0.398 to 0.791, *p* < 0.001) had statistically lower hypocalcemia whereas the BP ➔ RANKLi cohort (11.2% vs. 14.0%, OR: 0.775, 95% CI: 0.344 to 1.744, *p* = 0.5368) did not demonstrate a lower rate of hypocalcemia relative to the RANKLi cohort.

### 3.3 Chronic Pain Diagnosis and Opioid Use Patterns

Patient receiving bisphosphonates had significantly higher rates of opioid use (92.2% vs. 84.7%, OR: 2.14, 95% CI: 1.56 to 2.94, *p* < 0.001) compared to the overall BM group and comparable rates of chronic pain diagnosis (Table 3). Patients receiving RANKLi had significantly lower rates of opioid use (84.8% vs. 90.8%, OR: 0.56, 95% CI: 0.39 to 0.81, *p* < 0.001) and a non‐significant reduction in chronic pain diagnoses (50.8% vs. 55.9%, OR: 0.813, 95% CI: 0.65 to 1.01, *p* = 0.069) compared to the bisphosphonate group. With respect to the overall BM group, the subset of patients treated with RANKLi‐only had a comparable likelihood of opioid use despite an increased risk of chronic pain diagnosis, and the BP ➔ RANKLi group, also at increased risk of chronic pain diagnosis, had a statistically significant reduction in opioid use (91.6% vs. 81.3%, OR: 2.503, 95% CI: 1.083 to 5.787, *p* < 0.05) relative to the BP‐only group.

Cox proportional hazard model covariates, hazard ratios, 95% CI, and *p*‐values regarding opioid usage patterns are shown in Table 16. EBRT stands out as the most influential covariate increasing opioid usage across all cohorts. When comparing our BM cohort to the no BM cohort, application of EBRT correlated opioid usage in the BM cohort. Most notably, when comparing our BP‐only cohort to our RANKLi‐only cohort, EBRT was significantly associated with opioid usage in the BP‐only cohort (HR: 7.433, 95% CI: 1.038 to 53.24, *p* < 0.05). Similarly, when compared to the BP ➔ RANKLi cohort, the BP‐only cohort receiving EBRT showed a trend toward increased opioid usage receipt, although not statistically significant (HR: 5.501, 95% CI: 0.766 to 39.52, *p* = 0.0901).

**TABLE 16:** Cox proportional hazards of opioid usage associated with bone‐remodeling therapy.

	RCC + BM vs. No BM		RCC + BM vs. BP		RCC + BM vs. RANKLi		RCC + BM vs. RANKLi		RCC + RANKLi vs. BP à RANKLi		RCC + BP vs. BP à RANKLi	
	HR (95% CI)	*p*	HR (95% CI)	*p*	HR (95% CI)	*p*	HR (95% CI)	*p*	HR (95% CI)	*p*	HR (95% CI)	*p*
Male gender	1.652 (1.608, 1.696)	< 0.0001	0.747 (0.697, 0.801)	< 0.0001	1.216 (1.132, 1.306)	< 0.0001	1.658 (1.509, 1.821)	< 0.0001	0.991 (0.744, 1.143)	0.4594	1.529 (1.235, 1.893)	< 0.0001
Age	1.039 (1.025, 1.053)	< 0.0001	0.943 (0.901, 0.987)	0.0118	0.936 (0.894, 0.98)	0.0049	0.985 (0.893, 1.086)	0.7565	0.997 (0.871, 1.144)	0.9809	0.998 (0.875, 1.138)	0.9772
CKD	0.998 (0.997, 0.998)	< 0.0001	0.99 (0.988, 0.992)	< 0.0001	0.99 (0.988, 0.992)	< 0.0001	0.987 (0.983, 0.992)	< 0.0001	1.224 (0.98, 0.992)	< 0.0001	0.987 (0.982, 0.993)	< 0.0001
Tumor staging: S1	1.139 (1.12, 1.157)	< 0.0001	1.114 (1.06, 1.171)	< 0.0001	1.099 (1.046, 1.156)	0.0002	1.108 (1, 1.228)	0.0492	0.609 (0.911, 1.205)	0.5124	1.165 (1.017, 1.336)	0.028
Tumor staging: S2	1.084 (1.051, 1.117)	< 0.0001	1.074 (0.952, 1.212)	0.2479	1.074 (0.953, 1.211)	0.242	1.057 (0.81, 1.378)	0.6838	1.388 (0.725, 1.472)	0.8586	1.014 (0.68, 1.512)	0.946
Tumor staging: S3	1.097 (1.034, 1.164)	< 0.0001	1.145 (0.984, 1.332)	0.08	1.161 (1.001, 1.346)	0.0488	1.201 (0.906, 1.591)	0.2023	1.07 (1.066, 2.112)	0.0201	1.341 (0.911, 1.972)	0.1368
Tumor staging: S4	1.156 (1.098, 1.216)	< 0.0001	1.026 (0.909, 1.158)	0.674	0.998 (0.887, 1.124)	0.975	0.921 (0.724, 1.171)	0.5028	0.997 (0.588, 1.087)	0.153	1.04 (0.719, 1.504)	0.8344
Nephrectomy	0.669 (0.656, 0.683)	< 0.0001	0.876 (0.834, 0.92)	< 0.0001	0.895 (0.852, 0.94)	< 0.0001	0.843 (0.761, 0.933)	0.001	0.773 (0.791, 1.048)	0.1906	0.779 (0.681, 0.892)	0.0003
EBRT	1.461 (0.932, 2.291)	0.0987	1.895 (0.85, 4.223)	0.1179	1.722 (0.716, 4.142)	0.2248	7.433 (1.038, 53.24)	0.0458	—	—	5.501 (0.766, 39.52)	0.0901
Liver mets	1.188 (1.124, 1.255)	< 0.0001	1.109 (1.033, 1.19)	0.0041	1.133 (1.054, 1.217)	0.0006	1.27 (1.128, 1.43)	< 0.0001	1.714 (1.292, 1.795)	< 0.0001	1.212 (1.042, 1.41)	0.0128
Lung mets	1 (0.961, 1.04)	0.9885	1.007 (0.956, 1.06)	0.8034	0.989 (0.939, 1.043)	0.6858	1.165 (1.054, 1.287)	0.0028	1.157 (0.994, 1.31)	0.0615	1.227 (1.074, 1.403)	0.0027
Brain mets	0.936 (0.874, 1.002)	0.0554	0.91 (0.841, 0.984)	0.0186	0.909 (0.839, 0.986)	0.021	1.04 (0.908, 1.191)	0.5745	1.268 (0.855, 1.271)	0.6777	0.998 (0.84, 1.186)	0.986
Lymph nodes	1.137 (1.093, 1.183)	< 0.0001	1.03 (0.973, 1.091)	0.3055	1.044 (0.986, 1.106)	0.1419	1.018 (0.916, 1.131)	0.7441	1.223 (0.893, 1.204)	0.634	1.001 (0.871, 1.149)	0.9941

## 4 Discussion

Our analysis demonstrated, unsurprisingly, that patients with RCC and BM have worse outcomes than patients who do not develop BM. Because BM‐associated SREs are extremely painful, their development often results in severe quality of life deficits due to debilitating pain, prolonged immobility, and paralysis secondary to nerve compression [10]. In addition to pain medications, BPs and RANKLi are bone‐remodeling therapy agents that appear to have palliative effects on bone pain from BMs, potentially prolonging life while improving quality of life. Although bone‐modifying therapy use in treating BM for other malignancies has been well studied, their utility in RCC‐associated BM has not been thoroughly investigated to date [11]. Among the patients with BM, only 11.1% of patients received BPs and 10.8% of patients received RANKLi, indicating there is likely underutilization of bone‐remodeling agents for the treatment of BM in RCC, consistent with other data demonstrating low usage in patients with BM [12].

Current literature has demonstrated the effect of bone‐remodeling therapy on quality of life and pain management in the setting of tumor therapy‐induced osteoporosis, particularly in breast and prostate cancer due to hormonal withdrawal [13, 14, 15]. The use of bone‐remodeling therapy in treating RCC‐associated BM has not been investigated thoroughly and is not routinely practiced [16]. Notably, BPs have not been shown to confer any survival benefit, nor do they play any role in delaying the progression of metastatic lesions; their utility is limited to the prevention and/or delayed onset of SREs [16]. In addition to being renally excreted, BPs increase the risk of hypocalcemia, limiting the value of BPs in treating RCC‐associated BMs, in which renal dysfunction is commonplace [17]. BPs are primarily used as an adjunct to systemic therapies or radiotherapy and may exist as a purely palliative option for patients with advanced disease [16, 18]. This discrepancy in mortality and chronic pain likely reflects patients with severe BM and worse prognostic factors receiving bone‐remodeling treatment; our findings that additional metastatic lesions significantly contribute to the greater mortality in the BP‐only cohort corroborate this, making the improved survival seen in the RANKLi cohort intriguing.

Patients taking BPs may be transitioned to RANKLi for various reasons, particularly worsening renal function. To account for this, we propensity‐score matched our cohorts for CKD status/ESRD and nephrectomy status. Nephrectomy was found to be protective upon Cox proportional hazards analysis, likely reflecting advanced unresectable disease in those who did not undergo nephrectomy rather than an effect of drug therapy. Despite matching our cohorts for CKD and/or nephrectomy status, our BP ➔ RANKLi cohort had similar outcomes compared to those who only received RANKLi and significantly better 5‐year mortality and opioid usage rates compared to our BP‐only cohort. We attribute this perplexing finding to worse disease at the start of therapy. It seems reasonable to infer that those who started on BPs and later progressed to RANKLi would be more similar to the BP‐only cohort than to those who are untreated or received RANKLi only. If a consistent and profound baseline difference in disease severity exists between those initially receiving BPs compared to those initially receiving RANKLi, then the outcomes of those who started with BP and progressed to RANKLi therapy should be comparable to those only receiving BPs. Yet, this analysis showed improved survival for the BP➔RANKLi cohort, suggesting that the differences seen are not primarily due to baseline disease severity. Conversely, if the mortality differences between those receiving BPs and RANKLi are mainly due to social determinants of health (SDoH), the cohort starting on BPs would likely be more similar to the BP‐only group with comparable outcomes. Our data showed improved survival of RANKLi only and BP ➔ RANKLi groups compared to BP‐only, possibly indicative of an overall survival benefit of RANKLi therapy. This hypothesis should be evaluated prospectively but may be difficult given the relatively rarity of RCC BMs. Alternatively, subsequent initiation of RANKLi following BP therapy may reduce BM‐specific morbidity, opioid use, and hypercalcemia, thereby contributing to an improved survival not due to cancer‐specific effects.

RANKLi therapy strikingly appears to confer benefits compared to both the bisphosphonate and the baseline BM cohort. RANKLi had statistically significant improvements in median survival relative to the overall BM group and in 5‐year survival and opioid use rates relative to the bisphosphonate cohort. As these findings have not previously been published, we sought to control for confounders such as concomitant radiation therapy, variations in systemic therapies, and overall comorbidities to determine if this signal persisted and was not an artifact from underlying cohort differences. Within the limitations of the database to control for confounding effects, the survival advantage for RANKLi therapy remained significant. The consistent advantage led us to create a cohort to compare patients treated in sequence with BP then RANKLi. We expected that any undetected baseline differences in comorbidity or SDoH would mitigate the survival advantage seen in the RANKLi‐only group. Despite this, the BP ➔ RANKLi cohort demonstrated a significant survival advantage over BP alone. This was compelling since we believe most patients were switched from BP to RANKLi due to renal insufficiency, which has a well‐known association with inferior survival [19]. Thus, within the limitations of our ability to control for confounding variables, this difference remains consistent, suggesting that RANKLi therapy confers improved survival in patients with RCC + BM.

One possible explanation for this survival difference may be due to the interaction between RANKLi medications and Nuclear factor kappa B (NFkB) receptors, which have been found in both healthy and malignant bone tissues. In metastatic breast and prostate cancer patients, RANKLi have been shown to directly influence both primary and secondary tumorigenesis through NFkB inhibition, leading to decreased tumor growth and lower SRE rates in patients receiving RANKLi compared to those receiving BPs [20]. It is conceivable that this phenomenon holds true in RCC tumor deposits as well. Furthermore, RANK‐ligands have been implicated in systemic T‐lymphocyte modulation and M2‐macrophage recruitment. Immunomodulation in metastatic RCC has long been utilized to prolong survival with agents such as Interleukin‐2 (IL‐2) and Interferon‐α (IFN‐α) [21, 22]. In patients with RCC + BMs, RANKL inhibition has been shown to block regulatory T‐cell tolerance and enhance anti‐tumor lymphocytic responses, both independently and in combination with standard‐of‐care immunotherapy [5, 23]. The improved survival in patients treated at any time with RANKLi seen might be due to these types of direct immunological anti‐tumor effects, although much more investigation is needed before any conclusions may be drawn.

RANKLi are not without adverse effects, with significantly higher rates of hypocalcemia compared to both the overall BM and BP‐only cohorts, consistent with previously published literature. Studies report a 30‐day incidence of hypocalcemia nearing 35%; patients receiving RANKLi should be monitored carefully, particularly in patients with significant impairments to calcium homeostasis [24]. Fortunately, rates of osteonecrosis of the jaw (ONJ) and anaphylaxis, two life‐altering toxicities of both BPs and RANKLi, were negligible in every cohort, recapitulating prior studies demonstrating similarly low rates of both [25, 26]. The observed improvement in mortality within our RANKLi cohort may be attributable to several factors, including direct benefit from RANKLi therapy. However, patient selection may play a significant role, including worse disease states in patients receiving BP, exacerbation of renal dysfunction due to BP treatment, and various SDoH including insurance and socioeconomic status, which may directly affect patient outcomes and limit access to RANKLi therapy. We attempted to control for those factors measurable in TriNetX, but some are beyond the capture of a CPT/ICD10‐coded dataset. For example, RANKLi are costly, up to 100 times more expensive than bisphosphonates; consequently, patient insurance status may influence the choice of bone‐directed treatment. The improved outcomes for the BP ➔ RANKLi cohort seem to indicate that this may be more likely a result of medical therapy than non‐modifiable SDoH. Creating this cohort was our attempt to control for this disparity, but these factors are pervasive, profound, and mostly immeasurable in TriNetX. Although RANKLi therapy has demonstrated comparable incremental cost‐effectiveness ratios per quality‐adjusted life years to bisphosphonates in the treatment of osteoporosis, such data is unavailable in the setting of metastatic bone lesions [27]. TriNetX does not include insurance or reimbursement information, further limiting the scope of the dataset. Further studies are needed to better elucidate the cost‐effectiveness and equitable access of RANKLi in the treatment of RCC‐associated BMs.

We attempted to determine any signal indicating the influence of SDoH on our findings. We found that RANKLi are prescribed at lower rates in the Black and African American population compared to the white population, with respect to the representation of each race within the overall BM cohort (Table S1). This may suggest that some of the disparity in survival outcomes could well be due to SDoH rather than therapeutic effect. This racial disparity in RANKLi utilization has not been previously reported and should be the subject of further exploration in another database with more granular information on SDoH.

As noted previously, TriNetX has numerous limitations; TriNetX lacks data on several potential confounders such as patient insurance coverage, income bracket, and various socioeconomic demographics. Similarly, clinically useful patient stratification tools such as ECOG score, IMDC risk groups, and Karnofsky performance status are unavailable in TriNetX. Additionally, cancer‐specific mortality and granular data regarding total metastatic burden are unable to be ascertained from TriNetX, serving as additional caveats to the conclusions drawn from this manuscript. TriNetX has several other limitations common to all EMR‐based databases, including coding omissions, errors with data abstraction, and loss of patient data should they transfer care to a health system outside the TriNetX catchment [28]. Lastly, there is no data regarding dosage and scheduling of bisphosphonate therapy, particularly the need for dose modification due to diminished renal function [7]. Although we attempted to mitigate these concerns with PSM, certain key variables remain unaddressed.

## 5 Conclusion

In the setting of metastatic RCC, patients receiving RANKLi have significantly improved survival, decreased opioid usage, and lower SRE rates. Patients receiving BPs did not appear to have similar benefits in survival, pain, or SREs. The choice of bone‐remodeling class of therapy used may impact outcomes in patients with metastatic RCC and BMs. Further studies are warranted to elucidate the effects of socioeconomic demographics on RANKLi access and prescription patterns.

## Author Contributions

**Brian H. Im:** conceptualization (equal), data curation (lead), formal analysis (lead), investigation (lead), methodology (lead), writing – original draft (lead), writing – review and editing (equal). **Kevin K. Zarrabi:** conceptualization (equal), formal analysis (supporting), supervision (supporting), validation (lead), writing – review and editing (supporting). **Aaron R. Hochberg:** data curation (supporting), investigation (supporting), methodology (supporting), writing – review and editing (supporting). **Mihir S. Shah:** methodology (supporting), validation (supporting), writing – review and editing (supporting). **James R. Mark:** validation (supporting), writing – review and editing (supporting). **Joseph K. Izes:** validation (supporting), writing – review and editing (supporting). **Patrick T. Gomella:** validation (supporting), writing – review and editing (supporting). **Costas D. Lallas:** validation (supporting), writing – review and editing (supporting). **Leonard G. Gomella:** writing – review and editing (supporting). **Adam R. Metwalli:** conceptualization (lead), formal analysis (lead), investigation (lead), methodology (lead), project administration (lead), supervision (lead), validation (lead), writing – review and editing (lead).

## Conflicts of Interest

The authors declare no conflicts of interest.

## Supporting information


**Table S1:** Racial breakdown of individual cohorts.

## Data Availability

The data that support the findings of this study are available from the corresponding author upon reasonable request.
