# Supplementary material for: The Effects of Bone‐Remodeling Therapy on Survival, Pain, and Skeletal Related Events in the Setting of Renal Cell Carcinoma With Bone Metastases: A Multicenter Investigation From a Large Global Health Research Network (TriNetX)
Source: Cancer Med. 2026 Jan 14;15(1):e71133. doi: 10.1002/cam4.71133 (PMC12800899; doi:10.1002/cam4.71133)
Supplement: Supplementary file 1 — Table S1: Racial breakdown of individual cohorts. [file CAM4-15-e71133-s001.docx]

**Supplemental Table 1: Racial Breakdown of Individual Cohorts**

|  | White | Black or African American | Unknown Race | Asian |
| --- | --- | --- | --- | --- |
| RCC + BM | 75% | 8% | 14% | 3% |
| RCC No BM | 70% | 11% | 15% | 3% |
| BP | 75% | 7% | 13% | 3% |
| RANKLi | 81% | 4% | 10% | 3% |

RCC: Renal Cell Carcinoma

BM: Metastatic Bone Lesions

BP: Bisphosphonates

RANKLi: RANK Ligand Inhibitors
